# The relationship of PrEP beliefs to perceived personal, interpersonal and structural benefits and barriers to PrEP use in women who inject drugs

**DOI:** 10.1186/s12905-023-02452-7

**Published:** 2023-06-08

**Authors:** Kirsten Paulus, Patrick J.A. Kelly, Jesse Brajuha, Paul D’Avanzo, Emily F. Dauria, Aurora Trainor, Annabelle Alrez, Sarah Bauerle Bass

**Affiliations:** 1 Department of Social and Behavioral Sciences, Temple University College of Public Health, 1301 Cecil B. Moore Ave., Room 945, Philadelphia, 19064 USA; 2 Risk Communication Laboratory, Temple University College of Public Health, Philadelphia, USA; 3 Department of Behavioral and Community Health Sciences, University of Pittsburgh, 130 De Soto Street, Pittsburgh, 15261 USA

**Keywords:** HIV prevention, PrEP, Drug use, Gender, Key and vulnerable populations, Risk factors

## Abstract

**Background:**

Women who inject drugs (WWID) have significant biological, behavioral, and gender-based barriers to accessing HIV prevention services, including Pre-Exposure Prophylaxis (PrEP) medication. Little is known about how beliefs about PrEP impact both perceived barriers and benefits of PrEP use and how they may be related to the decision-making process.

**Methods:**

Surveys were conducted with 100 female clients of a large syringe services program in Philadelphia, Pennsylvania. The sample was categorized into three groups based on mean PrEP beliefs scores using terciles: accurate beliefs, moderately accurate beliefs, and inaccurate beliefs. Oneway ANOVA tests were used to compare groups by perceived benefits and barriers to PrEP, drug use stigma, healthcare beliefs, patient self-advocacy, and intention to use PrEP.

**Results:**

Participants had a mean age of 39 years (SD 9.00), 66% reported being White, 74% finished high school, and 80% reported having been homeless within the past 6 months. Those with the most accurate PrEP beliefs reported highest intent to use PrEP and were more likely to agree that benefits of PrEP included it preventing HIV and helping them “feel in charge”. Those with inaccurate beliefs were more likely to strongly agree that barriers, such as fear of reprisal from a partner, potential theft, or feeling they “might get HIV anyway”, were reasons not to use PrEP.

**Conclusions:**

Results indicate perceived personal, interpersonal and structural barriers to PrEP use are associated with accuracy of beliefs is, pointing to important intervention targets to increase uptake among WWID.

## Background

Injection drug use (IDU) is a significant route of HIV transmission in the U.S., the third most common attributable behavioral risk factor in incident HIV infections [1, 2]. In 2019, people who inject drugs (PWID) comprised 3% of the U.S. population but accounted for 7% of incident HIV infections [3]. Women who inject drugs (WWID) have added HIV risk; of the 7,000 new HIV diagnoses among women in the U.S. in 2019, 84% were attributable to heterosexual contact and 16% to injection drug use [4, 5]. Trends by sex indicate that while infections among men who inject drugs have remained stable over the past five years, infections in WWID have increased 7% [4, 5]. Without addressing HIV among WWID, it is estimated that one in every 23 will be diagnosed with HIV compared to one in every 36 men who inject drugs (MWID) [6].

Compared to their male counterparts, WWID have significant behavioral, and gender-based barriers to accessing available HIV prevention services that heighten their risk of HIV acquisition [2, 3, 7–9]. Additionally, among WWID, women who identify as transgender, racial and ethnic minorities, and young women are at an even greater risk [10]. Women’s HIV risk is often discussed based on childbearing and sexual behaviors, precluding attention to injection drug use [8]. But injection drug use and sexual behavior are concomitantly intertwined, and gender based [8]. Among heterosexual couples who use injection drugs, for example, men often exert control over women’s drug use and sexual behavior by controlling access to drugs and syringes or the actual preparation and injection process in which women are “second on the needle” and injected by their partners [10–12]. WWID also report being manipulated into sex work to support male partners, oftentimes engaging in unprotected sex, or having to negotiate the use of new injection paraphernalia [13, 14].

Despite the availability of effective HIV prevention methods, including pre-exposure prophylaxis (PrEP), the U.S. has not seen a significant decline in most groups at risk of HIV acquisition [2]. Clinical trials provide compelling evidence that PrEP can prevent or substantially reduce the risk of HIV acquisition when used correctly [14–16]. However, only 10% of women who could benefit from PrEP are currently on it [5]. Importantly, WWID are not included in a number of HIV prevention efforts as the main risk group (men who have sex with men and women who identify as transgender have been prioritized), resulting in them falling through the cracks of existing programs that do not address their unique needs as WWID [10, 13]. Current work has noted that perceived HIV susceptibility and perceived benefits of using PrEP are high among WWID but that specific barriers to uptake exist, including fear of side effects, HIV and IDU-related stigma, location of treatment, and psychological costs of patient-provider relationships [13, 17–19]. A recent study also notes a lack of engagement in healthcare services and largely male-targeted messaging around PrEP as other potential barriers [20].

Some recent studies have elucidated barriers of PrEP uptake in WWID that drive lower rates in PrEP use and how they are related to the decision-making process [21, 22]. Some of these barriers include sexual violence, economic insecurity, housing, instability, and syringe exchange program accessibility, as well as periods of perceived higher or lower HIV risk [21, 22]. Knowledge about these barriers is needed to help guide interventions to increase PrEP use in WWID, a significant at-risk population. The goal of this descriptive study, part of formative work to inform the development of a PrEP intervention to encourage PrEP uptake in WWID, was to identify potential factually inaccurate beliefs about PrEP and how that may be associated with other determinants of PrEP use, such as perceptions about barriers and benefits of PrEP use, including interpersonal and structural barriers such as drug use stigma and negative healthcare experiences, to better understand the decision-making process regarding PrEP use in WWID.

## Methods

### Setting, participants, and procedures

Data were collected as part of formative work for a National Institute on Drug Abuse funded study. Participants were self-identified female clients of Prevention Point Philadelphia (PPP), one of the largest syringe services programs for people who use drugs in the U.S. and located in the Kensington neighborhood, the epicenter of opioid use in Philadelphia. Offered services include a medical clinic providing medication assisted treatment, behavioral health and infectious disease prevention, syringe exchange, medical treatment, and housing services, to name a few [23].

Eligible participants: (1) Shared needles or had sex without a condom in the last month; (2) Were 18 years of age or older; (3) Spoke and read English; (4) Did not have HIV (based on self-report); (5) Were not using PrEP now or previously (self-report); and (6) Self-identified as a female client of PPP. Research staff approached women while they were receiving services at PPP and asked if they would be interested in taking a survey related to PrEP. Interested and eligible women were taken to a private area, such as a private medical exam room, for informed consent which was completed digitally through REDCap where they marked a box that they consented to participate in the study. They were then offered a hard copy of the consent for their records. Consented participants completed the survey with research staff on an iPad, in which research staff verbally administered the survey and entered responses into REDCap; participants were provided with a paper survey and laminated scale sheet to improve visibility and comprehensibility. Data collection occurred between August and December 2019. Participants received a $10 gift card upon completion. Temple University institutional review board reviewed and approved this study (#25,028).

### Measures

The survey was developed by the authors based on results from qualitative focus groups with WWID and in-depth interviews with PPP staff [24]. The survey consisted of sociodemographics, HIV-related items, and 64 scaled items. Sociodemographics included: race and ethnicity, age, highest level of education completed, employment, and housing status. Other items assessed sex work history, time in jail or prison, parole or probation status, health insurance status, perceived HIV risk, condom use, syringe sharing history, number of sexual partners, and familiarity with PrEP. Sexual behaviors (based on CDC risk categories) were created using some existing measures from prior literature as well as some created measures based on focus group results with the population of interest [25]. Based on the time at which this survey was administered, PrEP use was based on oral administration of a daily pill. HIV and PrEP knowledge were each assessed by a set of seven true/false questions and composite scores calculated (with 0 being incorrect and 1 correct). Intent to use PrEP was assessed by the question: “If your doctor asked you right now to decide about using PrEP, how do you think you would answer?” The response options were on a 0–10 Likert scale, with 0 as definitely would not want to use PrEP and 10 as definitely do want to use PrEP. The scaled items assessed PrEP perceptions, drug use stigma, and barriers to healthcare, separated into seven different blocks of statements [24]. Each item used a 0–10 Likert scale to assess agreement (0 = strongly disagree, 10 = strongly agree). The blocks were:

- **Beliefs about PrEP (10 items)**: Addressed common beliefs about PrEP, including it only being for certain subpopulations.
- **Benefits of Using PrEP (9 items)**: Included perceived benefits like ease of taking or protection during sex.
- **Barriers to Using PrEP (11 items)**: Included perceived barriers like side effects or safety/access when homeless.
- **Feelings about Getting Healthcare (10 items)**: Included barriers to getting healthcare like provider judgment about drug or sex life and feasibility of seeing a doctor.
- **Confidence in PrEP Knowledge (9 items)**: Included confidence in PrEP knowledge and trust in sources.
- **Unique Experience as Female-Identifying Drug User (9 items)**: Included unique issues to WWID like drug reliance, safety, and identity.
- **Confidence in Talking to Doctor (6 items)**: Included confidence in talking to a doctor and asking questions, based on Brashers et al.’s patient self-advocacy scale [26].

### Analytic plan

Analysis of variance (ANOVA) was used to evaluate associations between PrEP beliefs with each item related to perceived PrEP benefits and barriers within the six statement blocks. The sample (n = 100) was categorized based on the mean beliefs score from that statement block using terciles (Table 1). There was no missing data on any items of interest. Seven of the ten items measured inaccurate PrEP beliefs; the item that was worded “I am more likely to take PrEP if I am paid to take it” was determined to be an inaccurate belief about PrEP as payment to adhere to a medication is not sustainable and does not ensure correct adherence long term. The remaining items were reverse coded to reflect inaccurate beliefs. Sum scores of all ten items (range 0–10; minimum score = 0, maximum score = 90) were then calculated and put into three categories: (1) Accurate beliefs (n = 37, mean ≤ 15); (2) Moderately accurate beliefs (n = 34, mean 16–29); and, (3) Inaccurate beliefs (n = 29, mean $\ge$ 30). The baseline demographics of the three groups were compared using oneway ANOVA tests for continuous variables and Pearson *chi*-square tests for categorical variables. Oneway ANOVA tests were then used to compare the three belief groups to specific perceptions of benefits and barriers of PrEP.

**Table 1:** Survey items assessing PrEP beliefs

**Statement**	**Response Option**
PrEP is only for gay men.	0–10
PrEP is only for trans people.	0–10
PrEP is only for people from certain races/ethnicities.	0–10
PrEP sounds “too good to be true”.	0–10
PrEP makes people think they are invincible (like they can’t get HIV or other STI/STDs).	0–10
PrEP only protects against HIV, not other STDs.	0–10
PrEP is only for those who do sex work.	0–10
PrEP has a street value.	0–10
PrEP is safe and effective for women who inject drugs to use.	0–10
I am more likely to take PrEP if I’m being paid to take it.	0–10

## Results

### Sample demographics

The mean age of participants was 39 years (SD 9.00), 66% reported their race as Caucasian/White, 74% reported finishing high school or getting a GED, 6% were employed, and 25% were currently on parole or probation. 80% reported having been homeless within the past six months. Two significant differences were found between the three groups - ever hearing of PrEP (χ = 8.0237, *p =* 0.02) and intent to use PrEP (*F =* 4.17, *p =* 0.02) (see Table 2).

**Table 2:** Demographic and other characteristics of total analytic sample (n=100) and by group

Survey Item	Total (N = 100)	Group 1 (n = 37) accurate beliefs (≤ 15)	Group 2 (n = 34) moderately accurate beliefs (16–29)	Group 3 (n = 29) inaccurate beliefs (≥ 30)	p
Race/ethnicity^†^					0.79
African American/Black	17	7 (19%)	6 (18%)	4 (14%)	
Latino/a/x	11	3 (8%)	2 (6%)	6 (21%)	
White/Caucasian	66	26 (70%)	22 (64%)	18 (62%)	
Multi-racial/Multi-ethnic	5	1 (3%)	3 (9%)	1 (3%)	
Other	1	0 (0%)	1 (3%)	0 (0%)	
Age (mean, in years) ^‡^	39	39 (9.66)	37 (9.07)	40 (8.01)	0.49
Education^†^					0.13
Less than high school diploma or GED	26	5 (13%)	9 (26%)	12 (41%)	
Finished high school or got GED	35	15 (41%)	9 (26%)	11 (38%)	
Some College	21	8 (22%)	11 (32%)	2 (7%)	
Completed technical/vocational school or community college (associates degree)	13	6 (16**%)**	4 (12%)	3 (10%)	
College degree or above (bachelor’s degree or above)	5	3 (8%)	1 (3%)	1 (3%)	
Employed^†^	6	2 (5%)	3 (9%)	1 (3%)	0.66
Homeless past 6 mo^†^	80	34 (92%)	25 (74%)	21 (72%)	0.07
Ever exchanged sex for money/food/drugs/etc^†^	71	30 (81%)	22 (65%)	19 (66%)	0.23
Been to jail^†^	89	31 (84%)	33 (98%)	25 (86%)	0.17
Currently on parole/probation^†^	25	6 (16%)	11 (32%)	17 (59%)	0.41
Medically Insured^†^	86	31 (84%)	31 (91%)	24 (83%)	0.56
Condom use (yes)^†^	40	15 (41%)	13 (38%)	12 (41%)	0.97
Injected drugs past 3 mo^†^	64	26 (70%)	23 (68%)	15 (52%)	0.26
Suspected syringe share^†^	33	14 (38%)	13 (39%)	6 (21%)	0.99
Ever heard of PrEP	71	32 (86%)	23 (68%)	16 (55%)	0.02*
Number of sexual partners within past 3 mo (M, SD) ^‡^	13 (43)	20.42 (66.88)	8.18 (18.78)	9.00 (15.03)	0.43
Perceived HIV risk (1–10) (M, SD) ^‡^	2.2 (2.74)	1.75 (2.47)	2.59 (2.73)	2.31 (3.09)	0.43
HIV Knowledge Sum Score (0–10) (M, SD) ^‡^	4.96 (0.80)	4.84 (0.73)	4.85 (0.70)	5.24 (0.95)	0.08
PrEP knowledge Sum Score (0–10) (M, SD) ^‡^	5.57 (0.64)	5.68 (0.47)	5.62 (0.60)	5.38 (0.82)	0.05
Intent to Use PrEP (0–10) (M, SD) ^‡^	8.49 (2.17)	9.27 (1.41)	7.91 (2.65)	8.17 (2.11)	0.02*

^†^Chi square test

^‡^F-test

*p ≤ 0.05

### Perceptual variables

Reported means, F test results, and p-values are reported in Table 3 for all items. Significant results are described below. Means are listed in order by group (*accurate beliefs*, *moderately accurate beliefs*, *inaccurate beliefs)*.

**Table 3:** ANOVA results ? PrEP belief group by other statement blocks

Survey Item^†^	Group 1 (n = 37) accurate beliefs (≤ 15)		Group 2 (n = 34) moderately accurate beliefs (16–29)		Group 3 (n = 29) inaccurate beliefs (≥ 30)		*F*(2, 97)	*p*
	M	SD	M	SD	M	SD		
Benefits of using PrEP								
PrEP can keep me from getting HIV.**	9.02	2	8.29	3	6.41	4	6.47	0.002
Using PrEP would make me feel more in charge of my life.**	8.97	2	7.47	3	6.76	3	5.63	0.005
I would be able to enjoy sex more if I was taking PrEP.	7.84	3	6.91	3	6.90	3	1.05	0.36
PrEP is easy to take.	9.43	2	9.65	1	8.76	2	2.97	0.06
PrEP would not interfere with me injecting or taking drugs.*	9.00	3	9.35	2	7.32	4	3.41	0.04
I would not have to rely on my partner to use condoms if I was taking PrEP.	3.51	4	4.59	4	3.86	3	0.73	0.48
I would only have to take one pill a day if I was on PrEP.	9.46	1	8.56	3	8.62	2	1.99	0.14
PrEP would let me worry less about HIV and not feel guilty about having fun.*	8.84	2	7.29	3	7.35	3	3.80	0.03
PrEP is affordable.	8.68	2	8.35	3	7.69	3	1.09	0.34
Barriers to using PrEP								
It would be hard for me to take a pill every day.	1.65	3	3.18	3	2.21	3	2.17	0.12
Taking PrEP would dull my high.	0.77	2	1.12	2	2.53	3	3.00	0.06
Taking PrEP would cause too many side effects.*	1.65	3	1.91	2	3.45	3	3.78	0.03
I feel healthy so I don’t need to take PrEP.*	1.03	3	2.00	3	3.03	3	4.38	0.02
I already protect myself from HIV in other ways (i.e. condoms, not sharing needles).	3.81	4	4.06	3	5.14	4	1.23	0.30
Even if I take PrEP, I might get HIV anyway.*	3.76	4	5.82	4	5.17	3	3.25	0.04
My partner might hurt me if they knew I was on PrEP.***	0.11	0	1.85	3	2.28	3	7.60	< 0.001
I have a lot more worries in my life than getting HIV.***	1.00	3	4.56	4	3.55	4	10.02	< 0.001
If I started PrEP it would be hard to get to the doctor every three months.**	0.97	2	2.94	3	2.79	3	5.56	0.005
I don’t have a safe place to keep PrEP.***	0.62	2	3.06	3	3.03	3	8.45	< 0.001
I would be afraid if I had PrEP it would get stolen.**	1.02	3	3.26	3	3.34	4	5.94	0.004
Feelings about getting healthcare								
I don’t want to talk with a doctor about my drug use.	1.88	3	2.35	3	3.37	3	1.20	0.31
I don’t want to talk with a doctor about my sex life.	1.68	3	2.62	3	3.38	3	2.18	0.12
I am afraid I am judged by the doctor and other people who work in a doctor’s office (like front desk staff).	2.62	4	3.65	3	3.17	3	0.80	0.45
Doctors don’t want to treat people like me.	2.05	3	3.38	3	2.97	3	1.49	0.23
Getting a doctor’s appointment is easy.	6.59	4	6.03	4	5.48	3	0.76	0.47
It is easiest to go to the emergency room when I need to see a doctor.**	5.27	4	7.91	3	6.66	3	5.16	0.007
I have had positive interactions with the staff at most doctors’ offices or clinics (like nurses, aides, front desk staff).	7.27	4	7.09	3	6.45	3	0.47	0.63
I’m comfortable talking with doctors.	8.41	3	7.03	3	6.79	4	2.52	0.09
I feel doctors listen to me and do not rush me.	7.19	3	6.32	3	6.48	4	0.72	0.49
It’s hard to get to the doctor’s because it’s far away.*	2.65	4	4.79	3	4.00	3	3.25	0.04
Confidence about PrEP knowledge								
I understand how PrEP works.	8.95	2	8.21	3	7.51	2	2.62	0.08
I know where to get PrEP.	8.73	3	8.32	2	7.45	3	1.85	0.16
I have received enough education or counseling about PrEP.	8.21	3	7.38	3	6.55	3	2.75	0.07
I would trust PrEP information more if it came from a doctor.	4.43	4	5.36	4	5.76	3	1.09	0.34
I would trust PrEP information more if it came from my case manager.	3.51	4	4.62	4	5.52	3	2.35	0.10
I would trust PrEP information more if it came from my women friends.	3.27	4	3.62	4	4.72	3	1.32	0.27
I would trust PrEP more if it came from my male friends.	2.32	4	2.88	4	3.93	3	1.86	0.16
I would trust PrEP information more if it came from my drug using friends.	1.97	3	2.09	3	3.28	3	1.74	0.18
I would trust PrEP information more if it came from someone who is taking PrEP.	7.32	3	8.03	2	7.90	3	0.61	0.54
Unique experiences as a female drug user								
Most of my time is spent getting or using drugs.	6.23	4	7.46	3	6.53	4	0.81	0.45
My drug use keeps me from taking better care of my health.	7.04	4	7.73	3	7.37	3	0.28	0.75
I have psychiatric or psychological issues that get in the way of taking care of myself.**	3.16	4	5.68	3	5.79	4	6.05	0.003
I have to rely on a partner to survive.*	1.73	3	3.15	3	3.90	4	3.43	0.04
I am treated differently by people because I use drugs.	5.73	4	7.23	3	7.32	3	1.73	0.19
I feel confident in my ability to protect myself as a woman.	7.97	3	7.53	3	7.69	2	0.20	0.82
I do what is best for me regardless of what others think.	8.76	3	7.41	3	7.34	2	2.78	0.07
I am not like other women who inject or use drugs.	4.46	4	4.88	4	4.53	3	0.08	0.92
Sometimes I fall asleep or forget about things and can’t get things done (i.e. go to a doctor’s appointment).**	5.21	4	7.47	3	7.07	3	4.53	0.01
Confidence in talking to doctor								
I actively seek out information on my health.	7.37	4	6.12	4	6.27	3	1.35	0.26
I don’t get what I need from my doctor because I am not assertive enough.**	1.84	3	4.35	4	4.48	3	6.49	0.002
I am more assertive about my healthcare needs than most women who inject drugs.	6.43	4	6.38	3	6.45	3	0.00	1.00
If my doctor prescribes something I don’t understand or agree with, I question it.	8.86	2	8.32	2	7.83	2	2.01	0.14
If I am given a treatment by my doctor that I don’t agree with, I am likely not to take it.	7.24	4	7.59	3	6.52	3	0.81	0.45
I don’t always do what my doctor or health care worker asks me to do.	6.30	4	6.18	3	5.07	3	1.10	0.34
Intent to use PrEP in the future.*	9.27	1	7.91	3	8.17	2	4.17	0.02

^†^All item scores range 0–10

*p ≤ 0.05

**p ≤ 0.01

***p ≤ 0.001

### Benefits of using PrEP

Four significant differences were observed on benefits of using PrEP. The *accurate beliefs* group had higher mean scores compared to the other groups for the statements “PrEP can keep me from getting HIV” (*M* = 9.02 vs. *M* = 8.29 vs. *M* = 6.41, *F* = 6.47 (2,97), *p* = 0.002), “Using PrEP would make me feel more in charge of my life” (*M* = 8.97 vs. *M* = 7.47 vs. *M* = 6.76, *F* = 5.63 (2,97), *p* = 0.005) and “PrEP would let me worry less about HIV and not feel guilty about having fun” (*M* = 8.84 vs. *M* = 7.29 vs. *M* = 7.35, *F =* 3.80 (2,97), *p =* 0.03). However, for “PrEP would not interfere with me injecting or taking drugs,” those with moderately accurate beliefs had the highest score (*M* = 9.00 vs. *M* = 9.35 vs. *M* = 7.32, *F* = 3.41 (2,97), *p* = 0.04).

### Barriers to PrEP

Significant differences were found for eight out of the eleven items. For six of the items, the *accurate beliefs* group had lower mean scores than the *moderately accurate beliefs* and *inaccurate beliefs* groups for the following statements: “Even if I take PrEP, I might get HIV anyway” (*M* = 3.76 vs. *M* = 5.82 vs. *M* = 5.17, *F =* 3.25 (2,97), p = 0.04), “My partner might hurt me if they knew I was on PrEP” (*M* = 0.11 vs. *M* = 1.85 vs. *M* = 2.28, *F =* 7.60 (2,97), *p* = < 0.001), “I have a lot more worries in my life than getting HIV” (*M* = 1.00 vs. *M* = 4.56 vs. *M* = 3.55, *F =* 10.02 (2,97), p = < 0.001), “If I started PrEP it would be hard to get to the doctor every three months” (*M* = 0.97 vs. *M* = 2.94 vs. *M* = 2.79, *F* = 5.56 (2,97), *p =* 0.005), “I don’t have a safe place to keep PrEP” (*M* = 0.62 vs. *M* = 3.06 vs. *M* = 3.03, *F = 8.45* (2,97), p = < 0.001, and “I would be afraid if I had PrEP it would get stolen” (*M* = 1.02 vs. *M* = 3.26 vs. *M* = 3.34, *F =* 5.94 (2,97), p = 0.004). For one item, the *accurate beliefs* and *moderately accurate beliefs* groups had lower scores than for the *inaccurate beliefs* group: “I feel healthy so I don’t need to take PrEP” (*M* = 1.03 vs. *M* = 2.00 vs. *M* = 3.03, *F =* 4.38 (2,97), *p =* 0.04). Finally, the *accurate beliefs* group had the lowest score for one item: “Taking PrEP would cause too many side effects” (*M* = 1.65 vs. *M* = 1.91 vs. *M* = 3.45, *F =* 3.78 (2,97), *p =* 0.03).

### Feelings about getting healthcare

Two significant differences were found between groups. The *accurate beliefs* group had a lower mean score for both items: “It’s hard to get to the doctor’s because it is far away” (*M* = 2.65 vs. *M* = 4.79 vs. *M* = 4.00 *F =* 3.25 (2,97), *p =* 0.04) and “It is easiest to go to the emergency room when I need to see a doctor” (*M* = 5.27, *M* = 7.91, *M* = 6.66, *F* = 5.16 (2,97), *p* = 0.007).

### Confidence in PrEP knowledge

No significant differences were found in this block.

### Unique experience as female drug user

Three significant differences were found in this block. For all items, the *accurate beliefs* group had a significantly lower mean score: “I have psychiatric or psychological issues that get in the way of taking care of myself” (*M* = 3.16 vs. *M* = 5.68 vs. *M* = 5.79, *F = 6.05* (2,97), *p* = 0.003), “I have to rely on a partner to survive” (*M =* 1.73 vs. *M* = 3.15 vs. *M* = 3.90, *F* = 3.43 (2,97), *p* = 0.04), and “Sometimes I fall asleep or forget about things and can’t get things done” (*M* = 5.21 vs. *M =* 7.47 vs. *M* = 7.07, *F* = 4.53 (2,97), *p* = 0.01).

### Confidence in talking to doctor

One significant difference was found on the patient self-advocacy scale. The *accurate beliefs* group had the lowest mean score on the item: “I don’t get what I need from my doctor because I am not assertive enough” (*M* = 1.84 vs. M = 4.35 vs. M = 4.48, *F =* 6.49 (2,97), *p =* 0.002).

## Discussion

This study identified three distinct groups of WWID defined by how strongly they agreed with common beliefs about PrEP. Importantly, groups significantly differed from one another by their intent to use PrEP, with those in the *accurate beliefs* group indicating a very high intent to use PrEP if offered. This suggests future studies are needed to explore whether beliefs alone have an impact on PrEP intent and uptake. This is in line with Carter et al.’s recent study that indicated that education on the utility of PrEP in people who inject drugs was enough to change perceptions about their ability to use PrEP [18]. If future studies support this finding, correcting inaccurate beliefs might be an easy intervention target to encourage PrEP use. However, it is important to note that these findings did not assess specific structural barriers for WWID which may impact PrEP intention and uptake. More robust study designs could help better inform how beliefs, structural barriers, intention, and uptake are associated to develop interventions to improve PrEP uptake in WWID.

We also found that PrEP beliefs were most frequently associated with perceived barriers to use. Items related to having other more important worries and not having a safe place to keep PrEP illustrated that perhaps those who agreed with inaccurate beliefs about PrEP may also perceive more structural barriers to PrEP uptake and may not see HIV prevention as a priority. However, structural barriers were not explicitly evaluated in this study beyond perceptions and need further exploration. This prioritization of other things, such as housing, relationships, or drug use, is also seen in a recent study by Flesher et al., indicating an important intervention target [27]. Structural level interventions that address housing and economic resources as well as individual level targets may be needed to adequately address PrEP use barriers in WWID, many of whom are currently people who are experiencing homelessness. Additionally, participants highlighted a connection between beliefs about PrEP and their willingness to use PrEP, as women who did not believe PrEP was effective or easy to take were also less likely to say they would use it. Although our sample size is not big enough to adjust for potential confounders, it provides some indication that those who strongly agreed with common inaccurate beliefs about PrEP were also more likely to identify stronger with potential negative consequences of PrEP use.

Another important finding from this descriptive study was the relationship of PrEP beliefs with personal psychosocial factors, such as experiences being a woman who uses drugs, experiences and confidence in getting healthcare and the ability to self-advocate in a healthcare setting. Our preliminary results indicate another potential relationship that must be explored further: those with *inaccurate beliefs* may be more likely to believe they have healthcare access barriers, potentially due to negative lived experiences and lower confidence in being able to self-advocate in the healthcare setting. As Biello et al. note, understanding not only personal barriers to PrEP use but interpersonal and clinical/structural barriers is important to gain a more nuanced understanding of how PWID, and WWID specifically, may be thinking about using PrEP as an HIV prevention method [27]. This study is the first to note the potential relationships among these different levels of barriers as perceived by WWID, an important contribution to understanding how best to encourage PrEP use among this population for future interventions.

Additionally, our findings also suggest that it is possible *inaccurate PrEP beliefs* may dominate within communities that are more unsure about their access to traditional healthcare and resort to emergency care as needed, which may reduce the amount, quality, and accuracy of HIV prevention information [18]. However, further research is needed to verify this relationship. For those with *inaccurate beliefs*, reliance on emergency room care could impede them from seeing a regular healthcare provider routinely. In turn, this may prevent them from establishing a relationship with a healthcare provider and having access to educational resources about PrEP and to PrEP itself in a one-on-one setting. Emergency rooms have great potential to be used as a viable catalyst for connecting WWID to PrEP, but due to current long wait times and patient volume, this opportunity is suboptimal [28]. It should also be noted that while Philadelphia is a large urban center with plenty of places to access healthcare, because of drug use stigma and other structural barriers WWID will often use PPP medical services as their first line of care. In fact, although at the time this study was done PrEP distribution was new, PrEP has become an important service at PPP, with specific staff hired to provide PrEP counseling. Results from this study have been used by agency staff to better develop their PrEP services specifically for women. Thus, implementing emergency room and syringe exchange program-based routine HIV screening and sexual health counseling on PrEP may improve access to PrEP and associated resources.

Associated with negative healthcare experiences were overall feelings of stigma and mental health issues experienced by WWID, which may also impact healthcare use. In this sample those with *inaccurate* or *moderately inaccurate beliefs* reported suffering disproportionately from psychiatric or psychological issues. Additionally, these groups shared that they oftentimes were unable to do things like go to a doctor’s appointment or take a pill every day. We hypothesize that this may connect to their willingness or desire to seek out healthcare or use PrEP, as they fear being stigmatized or judged for their drug use; however, this study alone cannot draw causal conclusions about this relationship. [13, 29–33]. Previous work has shown that WWID do not want to discuss their personal drug use behavior, and thus do not disclose their drug use to health professionals due to fear of what others may think [12]. This may keep WWID from asking for or being recommended PrEP, despite their HIV risk. Additionally, WWID may be dissuaded from using PrEP if their partner does not want them to use it or if they are experiencing gender-based or interpersonal violence and thus fear seeking out any healthcare at all. This was suggested by our study, with those with *inaccurate* or *moderately inaccurate beliefs* reported sometimes having to rely on their partner to survive [13, 19, 34, 35]. Even if they do attempt to seek out care, compared to MWID, WWID report experiencing much higher rates of stigma at clinics, which may be compounded by mental health stigma if there are underlying psychological or psychiatric issues [15].

Overall, understanding the associations between beliefs about PrEP and other perceptual variables provides context for establishing how best to target PrEP messages to WWID, and specifically to sub-groups who may have less accurate understanding of PrEP. Being able to characterize WWID by their PrEP beliefs could enable the development of more targeted intervention strategies, addressing personal, interpersonal, and structural barriers to uptake. Interventions that incorporate peer support to correct inaccurate beliefs, for example, could address both personal and interpersonal barriers, empowering WWID to seek out PrEP. In addition, if WWID believe that PrEP can help them prevent HIV and access to healthcare that they believe will not be stigmatizing is improved, PrEP use may be more normalized. However, at this moment it is uncertain whether an intervention targeting PrEP beliefs would impact PrEP uptake also addressing structural barriers that impact access or ability to easily use PrEP as part of the intervention. Future research is necessary, as well as evaluating actual PrEP uptake and its association with PrEP beliefs, to ensure that an intervention motivated by beliefs about PrEP is the best tactic.

There are some limitations to the study. Because of the nature of cross-sectional data, temporality inferences cannot be made. The survey also relies on self-reported data, so it is possible that responses may be subject to social desirability bias. Results may also not be generalizable to the broader WWID population because it was conducted only in Philadelphia and among women who are currently clients of a large social services agency that provides PrEP. Our sample size is relatively small, although it is broadly in line with similar studies and populations, even though this poses a limitation to using ANOVA in our analyses [21, 22] and limited our ability to adjust for potential confounders. Those in other geographic areas or countries who may not have access to these services may hold different beliefs about PrEP and PrEP use. Because of the timing of this survey, PrEP was also only available as an oral, daily medication. Attitudes about newer modalities (i.e. injectable PrEP) could change perceptions. Finally, categorizing the belief item “I am more likely to take PrEP if I am paid to take it” as inaccurate has potential implications for the three identified groups. This item is not explicitly inaccurate, but rather represents a perspective that dissuades PrEP use in the real world. Compared to the other inaccurate belief statements, this does not represent factual inaccuracy but rather a belief that could negatively impact PrEP uptake.

## Conclusions

Although a small descriptive study, results have the potential to inform future conceptual frameworks and intervention development to increase PrEP uptake among WWID to improve accurate communication about HIV risk and PrEP. Addressing personal, interpersonal and structural barriers, and how they are associated with accuracy of PrEP beliefs, is a novel way to understand how to effectively address poor PrEP uptake in this at-risk community.

## Data Availability

The datasets generated and/or analyzed during the current study are not publicly available due to privacy restrictions but are available from the corresponding author on reasonable request.

## List of abbreviations

WWID: Women who inject drugs
PWID: people who inject drugs
IDU: injection drug use
PrEP: pre-exposure prophylaxis
PPP: Prevention Point Philadelphia
ANOVA: analysis of variance test
